# Mechanical, Durability, Carbon Footprint, and Economic Assessment of Sand–Gravel–Gneiss Aggregate Mixtures for Sustainable Road Construction

**DOI:** 10.3390/ma19173679

**Published:** 2026-08-29

**Authors:** Agnieszka Nowaczek, Joanna Kulczycka, Marek Bęben, Dariusz Kasperek, Zygmunt Kowalski, Agnieszka Makara, Natalia Generowicz-Caba

**Affiliations:** 1 Mineral and Energy Economy Research Institute, Polish Academy of Sciences, Wybickiego 7a, 31-261 Krakow, Poland; 2 Faculty of Management, AGH University of Krakow, Gramatyka 10, 30-067 Krakow, Poland; 3 Awida Sp. z o.o., Brzegowa 1A, 55-200 Bystrzyca, Poland; 4 Faculty of Chemical Engineering and Technology, Cracow University of Technology, Warszawska 24, 31-155 Krakow, Poland

**Keywords:** sand–gravel–gneiss mixtures, crushed aggregates, road construction materials, mechanical performance, California Bearing Ratio (CBR), carbon footprint assessment, total cost of ownership

## Abstract

This study evaluates the influence of different crushed gneiss contents on the mechanical, durability, environmental, and economic performance of sand–gravel/gneiss mixtures for sustainable road construction. Natural sand–gravel aggregate was blended with crushed gneiss at three proportions (10%, 30%, and 50% by mass). The experimental program included grain size analysis, compaction characteristics, California Bearing Ratio (CBR), deformation modulus, and freeze–thaw durability tests. Environmental performance was assessed using a cradle-to-site carbon footprint approach based on Life Cycle Assessment principles according to ISO 14040, ISO 14044, and ISO 14067, with the analysis focused on Global Warming Potential (GWP100), combined with a Total Cost of Ownership analysis. Increasing gneiss content improved mechanical performance, with CBR increasing from 45% to 95% and deformation modulus from 110 to 185 MPa. The 70/30 sand–gravel/gneiss mixture provided the most balanced performance among the investigated compositions, combining high bearing capacity, satisfactory freeze–thaw resistance, and favorable environmental and economic characteristics. Its carbon footprint was 3.90 kg CO_2_ eq./t, with diesel consumption during aggregate handling identified as the dominant emission source (69%), followed by gneiss transportation (20%) and electricity consumption (12%). Higher gneiss contents and longer transport distances increased environmental impacts and production costs. The results indicate that selecting an appropriate aggregate composition and reducing transport-related emissions can support lower-carbon construction materials while maintaining required engineering performance.

## 1. Introduction

Mineral aggregates are fundamental materials in road construction and constitute the largest volume of raw materials used in transport infrastructure worldwide. They account for approximately 70–80% of asphalt pavements and up to 90% of cement concrete pavements; therefore, their physical, mechanical, and hydraulic properties largely determine pavement strength, durability, and service life [1,2,3,4]. The continuously growing demand for transport infrastructure has resulted in a global aggregate consumption exceeding 40 Gt annually, making aggregates one of the most intensively exploited natural resources [5,6,7,8]. At the same time, increasing environmental awareness and climate policies have shifted attention toward improving resource efficiency and reducing the embodied carbon of construction materials.

Natural aggregates, including sand and gravel, are widely used because of their availability, relatively low processing requirements, and favorable compaction characteristics [9,10,11]. Their rounded particle shape promotes workability and permeability, making them suitable for embankments, frost protection layers, drainage systems, and pavement subbases. However, the limited interlocking between rounded particles reduces their resistance to permanent deformation and restricts their application in heavily loaded pavement structures [12,13,14]. Conversely, crushed aggregates produced from hard rocks, such as gneiss, exhibit angular particle geometry, high mechanical strength, excellent abrasion resistance, and superior frost durability, enabling their use in pavement base courses and asphalt mixtures subjected to high traffic loads [15,16,17]. These advantages, however, are accompanied by higher energy consumption during extraction, crushing, and processing, as well as increased transportation requirements when suitable rock deposits are located far from construction sites.

Aggregate selection has therefore become a compromise between engineering performance, economic feasibility, and environmental impact. Recent developments in sustainable pavement engineering increasingly emphasize the efficient use of locally available resources while minimizing the consumption of high-quality crushed aggregates and reducing greenhouse gas emissions. Blending natural sand–gravel aggregates with crushed rock has emerged as a promising strategy to combine the favorable compaction and drainage properties of natural materials with the superior load-bearing capacity of crushed aggregates [18,19,20]. Such mixtures can improve pavement performance while reducing the demand for energy-intensive crushed rock, contributing to more sustainable resource management.

The engineering properties of both aggregate types have been extensively investigated. Natural sand–gravel aggregates generally exhibit good compactability, relatively high hydraulic conductivity, and satisfactory bearing capacity after proper compaction, although excessive fines may reduce permeability, frost resistance, and mechanical performance [21,22,23]. Crushed gneiss aggregates are characterized by high compressive strength, low water absorption, excellent resistance to fragmentation, and high freeze–thaw durability [24,25,26]. Their quality is commonly assessed using parameters such as Los Angeles abrasion resistance, particle density, water absorption, and polishing resistance, which determine their suitability for heavily trafficked pavements [27]. Because of these complementary characteristics, mixtures of natural and crushed aggregates have attracted increasing interest as a means of optimizing pavement material performance [28,29,30,31].

The mechanical behavior of unbound aggregate mixtures depends not only on the intrinsic properties of aggregate particles but also on their morphology, arrangement, and internal structure. Particle angularity, surface texture, shape, and size distribution influence interparticle contact, frictional resistance, and mechanical interlocking within the granular skeleton, thereby affecting stiffness, bearing capacity, and resistance to permanent deformation [19,32]. Crushed aggregates with angular particles and rough surfaces can enhance load-bearing capacity by improving particle interlocking, confinement, and stress transfer between individual grains [19]. In contrast, rounded natural aggregates generally provide better workability and drainage characteristics but may exhibit lower resistance to deformation due to reduced mechanical interlocking [19,32]. The performance of granular mixtures is also influenced by particle packing, void structure, and aggregate gradation, as an appropriate distribution of particle sizes promotes denser packing, increases interparticle contact, and improves stress transfer after compaction [32,33]. Moreover, compaction conditions play an important role in developing mechanical strength, since higher dry density reduces void space and enhances the ability of the granular skeleton to transfer applied loads [33]. The content and characteristics of fine particles may further affect moisture sensitivity, permeability, and strength development by modifying the internal pore structure and interactions between coarse and fine fractions [33,34]. Therefore, the combination of rounded sand–gravel aggregates with angular crushed gneiss may provide a favorable balance between compactability, drainage characteristics, and load-bearing capacity; however, the resulting performance depends on the interaction between particle morphology, mixture composition, gradation, and compaction conditions.

Despite extensive research on natural and crushed aggregates individually, systematic investigations of controlled sand–gravel–gneiss mixtures remain limited. In particular, the combined effects of crushed gneiss content, particle morphology, aggregate gradation, and compaction conditions on bearing capacity, deformation characteristics, permeability, and frost durability under comparable testing conditions are still insufficiently understood. Furthermore, previous studies have focused mainly on engineering performance, while the environmental and economic aspects of aggregate mixture design, particularly under real production conditions, have received considerably less attention [35,36,37].

Alongside technical performance, the environmental footprint of aggregate production has become an increasingly important criterion in construction material selection. Although natural aggregates require substantially less processing than materials such as cement or steel, their production still involves energy consumption associated with excavation, loading, screening, stockpiling, and transportation. Diesel-powered mobile equipment is commonly identified as one of the major sources of greenhouse gas emissions in aggregate production, while transportation may substantially increase the embodied carbon because aggregates have high density and relatively low economic value per unit mass [37,38]. Consequently, transportation distance often plays a critical role in determining both the environmental and economic efficiency of aggregate supply chains.

Life Cycle Assessment (LCA) has become a widely applied methodology for evaluating the environmental performance of construction materials and is standardized by ISO 14040, ISO 14044, and ISO 14067 [39,40,41]. Numerous studies have demonstrated the usefulness of LCA for evaluating construction materials; however, many assessments rely on generic databases and average emission factors that may not fully represent specific quarry conditions, operational practices, or transportation scenarios. A recent study has further confirmed the applicability of LCA and Life Cycle Impact Assessment (LCIA) for evaluating sustainable construction materials and supporting the selection of alternative technological solutions. For example, LCA/LCIA approaches have been applied to assess the environmental feasibility of replacing conventional Portland cement with coal gangue-based geopolymer materials, demonstrating their value in comparative sustainability assessments of construction materials [42]. The use of site-specific operational data can improve the reliability of carbon footprint assessments and provide more realistic information for practical decision-making [43,44,45].

The environmental performance of aggregate mixtures is strongly influenced by their composition. Increasing the proportion of crushed aggregate generally improves mechanical performance but may simultaneously increase emissions associated with quarrying, processing, and transport. Therefore, selecting a well-balanced aggregate composition requires consideration of engineering performance together with environmental and economic aspects. In recent years, the integration of Life Cycle Assessment with economic approaches such as Total Cost of Ownership (TCO) or Life Cycle Costing has gained increasing attention because it enables simultaneous evaluation of environmental burdens and production costs, supporting more sustainable infrastructure planning [44,46,47].

Despite the growing interest in sustainable construction materials, comprehensive studies integrating laboratory characterization, carbon footprint assessment, and economic evaluation of sand–gravel–gneiss mixtures based on real production data remain scarce. In particular, limited information is available on how varying crushed gneiss content affects both the engineering properties of aggregate mixtures and their environmental and economic performance under representative production and transportation conditions.

The environmental performance of aggregate mixtures is strongly influenced by their composition. Incorporating higher proportions of crushed aggregate generally improves mechanical performance but simultaneously increases emissions associated with quarrying, crushing, and transport. Therefore, identifying a balanced aggregate composition requires considering engineering performance together with environmental and economic aspects. In recent years, the integration of Life Cycle Assessment with economic approaches such as Total Cost of Ownership (TCO) or Life Cycle Costing has gained increasing attention because it enables simultaneous evaluation of environmental impacts and production costs, supporting more sustainable infrastructure planning [44,46,47].

Despite the growing interest in sustainable construction materials, comprehensive studies combining laboratory characterization, environmental assessment, and economic evaluation of sand–gravel–gneiss mixtures based on real production data remain scarce. In particular, limited information is available on how varying crushed gneiss content influences both the engineering properties of aggregate mixtures and their carbon footprint under representative production and transportation conditions.

Therefore, this study evaluates the influence of crushed gneiss content on the mechanical, environmental, and economic performance of sand–gravel–gneiss aggregate mixtures intended for sustainable road construction [48]. The objective was to determine how the proportion of crushed gneiss affects the mechanical performance, durability, environmental impact, and economic aspects of the investigated mixtures, and to identify the composition showing the most balanced performance among the evaluated variants. Natural sand–gravel aggregate was blended with crushed gneiss at proportions of 10%, 30%, and 50% by mass and investigated under identical laboratory conditions. Mechanical properties, including grain-size distribution, compaction characteristics, California Bearing Ratio (CBR), deformation modulus, hydraulic conductivity, abrasion resistance, and freeze–thaw durability, were assessed. In addition, a cradle-to-site Life Cycle Assessment (LCA), focused on carbon footprint assessment according to ISO 14067, and a Total Cost of Ownership (TCO) analysis based on site-specific operational data were performed to quantify greenhouse gas emissions and production costs and to evaluate the influence of mixture composition and transportation requirements.

## 2. Materials and Methods

### 2.1. Materials

**Figure 1:**
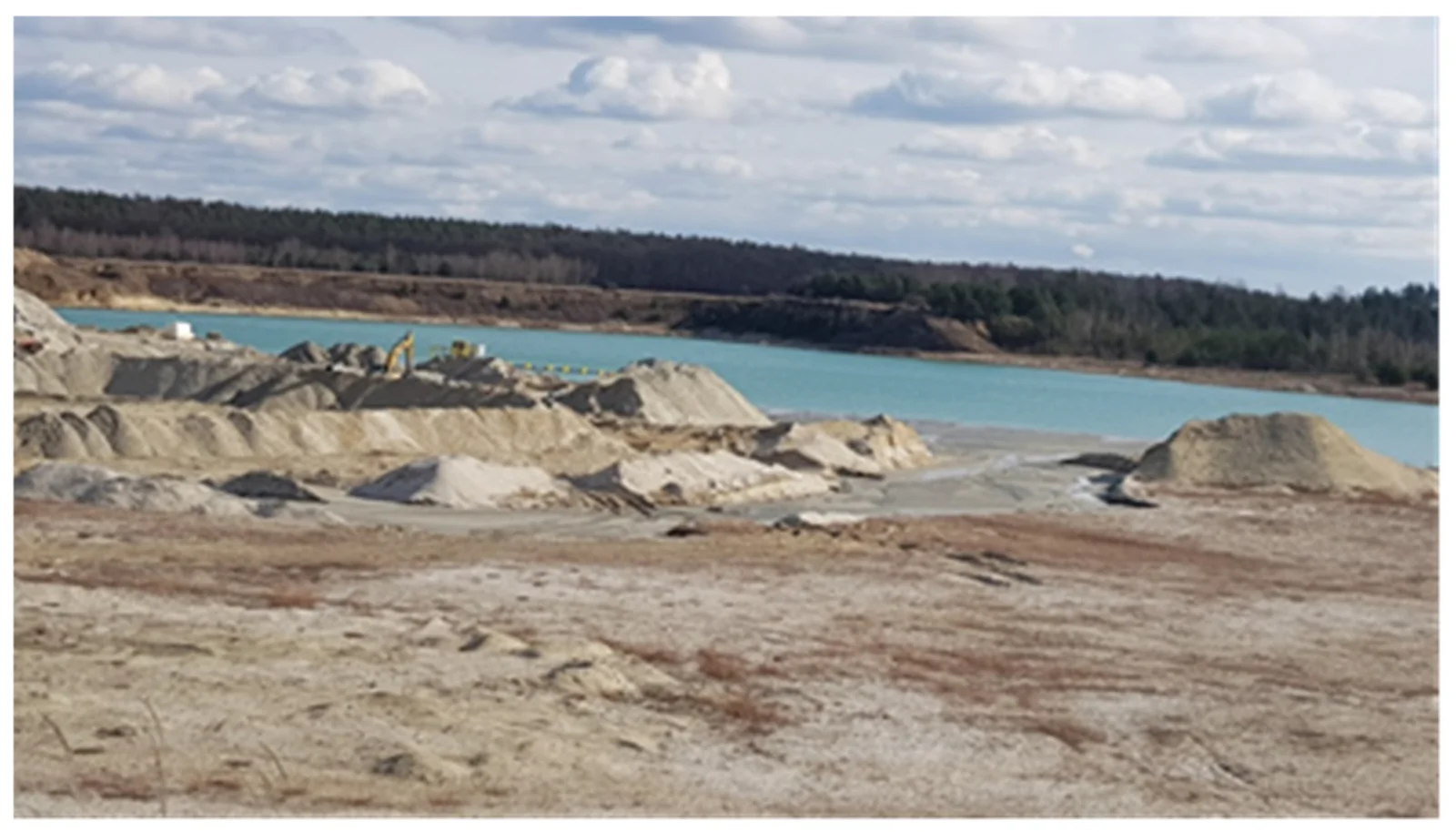
AWIDA sand mine in Bystrzyca Oławska.

The AWIDA sand mine (Figure 1) is located in Bystrzyca near Oława, Lower Silesian Voivodeship, Poland. The company has operated since 1990. Sand is extracted both from dry workings and from the bottom of a flooded excavation [49]. According to the national mineral-resources inventory [50], the deposit contains geological resources of approximately 16 million t and industrial resources of approximately 6 million t. The AWIDA facility produces excavated and washed sand as well as natural sand–gravel aggregate (pospółka).

In the present study, waste gneiss aggregate originating from the Dobroszowice quarry was used as a crushed aggregate component [43]. The material was selected due to its suitability for incorporation into mineral mixtures intended for road construction applications. Gneiss from the Dobroszowice deposit is characterized by favorable physicomechanical properties, including low porosity (0.81%), low water absorption (0.45%), high compressive strength (approximately 139 MPa), and high resistance to abrasion (Deval abrasion coefficient: 4.08%) [51]. The rock exhibits satisfactory frost resistance, which confirms its suitability as a durable aggregate component in construction applications [51].

The chemical composition of gneiss from the Dobroszowice 1 deposit shows natural variability. According to Kania et al. [51], the content of the main chemical components ranges as follows: SiO_2_ 65.48–72.98 wt.%, Al_2_O_3_ 10.02–19.11 wt.%, K_2_O 0.41–6.91 wt.%, Na_2_O 2.41–7.03 wt.%, Fe_2_O_3_ 1.50–4.12 wt.%, CaO 0.68–2.91 wt.%, MgO 0.20–1.23 wt.%, and TiO_2_ 0.11–0.60 wt.%. The mineral composition is dominated by quartz, feldspars, and micas, which determine the mechanical properties and weathering resistance of the material [51].

For the crushed aggregate used in the present study, technical data provided by the material supplier indicate a grain density of 2.64–2.67 Mg/m^3^, water absorption WA24 of approximately 0.4%, and resistance to fragmentation corresponding to LA category ≤ 16. The aggregate was processed to obtain a particle-size distribution within the range of 0–31.5 mm.

**Figure 2:**
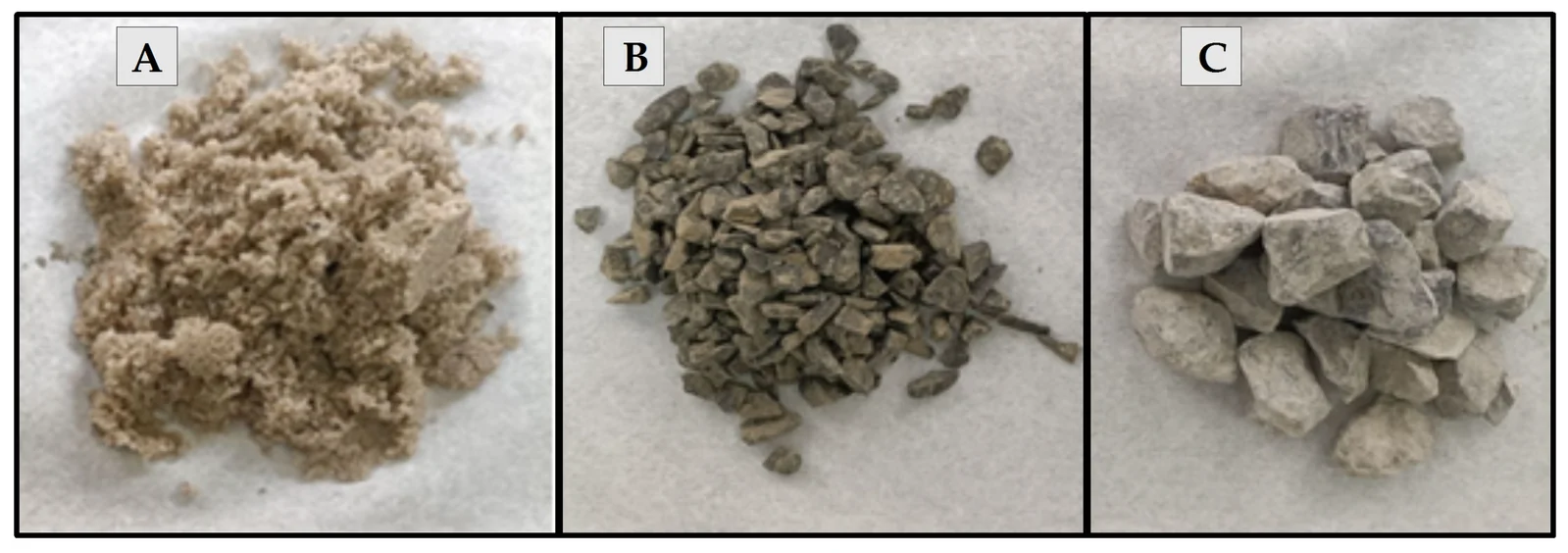
Images of aggregates used in the study: (**A**) natural sand–gravel aggregate (pospółka), (**B**) crushed gneiss aggregate (2–8 mm), (**C**) crushed gneiss aggregate (8–16 mm).

Gneiss and sand–gravel aggregates used in this study (Figure 2) were:

- Crushed gneiss aggregate, obtained from the Dobroszowice quarry and characterized by angular particles and a rough surface texture. The angularity of crushed particles may enhance mechanical interlocking within the granular structure.
- Natural sand–gravel aggregate (pospółka), obtained from the AWIDA facility and consisting of rounded particles with continuous gradation within the range of 0–31.5 mm.

The natural sand–gravel aggregate used in this study was characterized by a balanced particle-size distribution, supporting suitable compaction characteristics and drainage behavior. Its approximate composition was:

- 50% sand fraction, contributing to mixture homogeneity and workability;
- 40% gravel fraction, forming the main load-bearing skeleton of the material;
- 10% clay fraction, which may influence moisture sensitivity, permeability, and compaction behavior of the aggregate mixture.

The clay content was an inherent characteristic of the natural sand–gravel aggregate obtained from the AWIDA facility and remained constant in all investigated mixtures. Therefore, the comparative analysis focused on the influence of crushed gneiss content while maintaining the same natural aggregate source.

The main physical characteristics of the natural sand–gravel aggregate were as follows:

- Bulk density: 1.8 t/m^3^;
- Hydraulic conductivity: 1 × 10^−4^ m/s, determined according to Darcy’s law;
- Optimum moisture content for compaction: approximately 5%.

### 2.2. Experimental Processing Line and Materials

The experimental investigation was carried out using a quarter-scale pilot processing line designed to simulate aggregate mixture preparation under controlled laboratory conditions. The installation was developed to evaluate mixtures of natural sand–gravel aggregate and crushed gneiss aggregate intended for road construction applications.

The processing system consisted of two storage hoppers with a capacity of 500 kg each. The hoppers were adapted from previously used gypsum storage units. Raw materials were transported from the hoppers to a weighing station using screw conveyors equipped with frequency-controlled electric drives, enabling controlled feeding and accurate dosing of individual aggregate components while minimizing material losses during batching.

A digital weighing system with a maximum capacity of 500 kg was used for dosing individual aggregate fractions. The system enabled precise control of the mass proportions of individual components and recording of the obtained measurement data. Mixing was performed in a conventional concrete mixer with a working capacity of approximately 150 kg per batch. The mixing time was adjustable between 3 and 10 min, while the rotational speed ranged from 20 to 50 rpm. The same preparation conditions were applied for all investigated mixtures to ensure comparability of the results.

Natural sand–gravel aggregate was obtained from the AWIDA processing facility (Bystrzyca Oławska, Poland), whereas crushed gneiss aggregate originated from the Doboszowice quarry. The natural sand–gravel aggregate constituted the main component of the investigated mixtures, while crushed gneiss aggregate was introduced as a complementary component. The investigated mixtures differed in the proportion of crushed gneiss aggregate, enabling evaluation of the influence of aggregate composition on the mechanical, environmental, and economic performance of the mixtures.

All experimental tests were performed using two independent specimens for each investigated mixture under identical preparation conditions. The specimens were prepared and tested under identical conditions to ensure repeatability of the experimental procedure. The reported values represent the average results obtained from two measurements.

### 2.3. Mixture Design

Three aggregate mixtures were prepared [48] by varying the proportion of crushed gneiss aggregate while maintaining the total aggregate mass constant. The investigated compositions were selected to represent different levels of gneiss incorporation into the natural sand–gravel aggregate matrix:

- M1: 90% natural sand–gravel aggregate + 10% crushed gneiss aggregate;
- M2: 70% natural sand–gravel aggregate + 30% crushed gneiss aggregate;
- M3: 50% natural sand–gravel aggregate + 50% crushed gneiss aggregate.

The selected mixture proportions were based on the assumption that natural sand–gravel aggregate provides favorable compaction and hydraulic characteristics, while the incorporation of crushed gneiss aggregate may modify the granular structure due to differences in particle morphology and mechanical properties. The experimental design enabled comparative evaluation of the influence of crushed gneiss content on the mechanical performance, durability-related properties, environmental impact, and economic aspects of the investigated aggregate mixtures.

### 2.4. Sample Preparation

The production procedure consisted of three consecutive stages: material dosing, mixing, and laboratory sampling.

First, individual aggregate fractions were discharged from the storage hoppers and weighed according to the required mixture composition using the electronic weighing system. The batch masses were recorded by the data acquisition system.

The weighed materials were subsequently transferred into the concrete mixer and blended for approximately 5–7 min at a moisture content of approximately 5% and a rotational speed of about 35 rpm. These mixing conditions were applied to ensure homogeneous distribution of the aggregate fractions and minimize the risk of segregation.

Following mixing, representative samples were collected for laboratory characterization and durability-related testing. All samples were prepared according to the same procedure to maintain comparable conditions among the investigated mixtures.

### 2.5. Laboratory Testing

The engineering performance of the investigated mixtures was evaluated using laboratory procedures commonly applied for the characterization of unbound aggregate mixtures intended for civil engineering works and road construction. The evaluation was performed considering the requirements applicable to aggregates used in unbound and hydraulically bound materials according to PN-EN 13242+A1:2010 [28]. All laboratory tests were performed using two independent specimens for each investigated mixture under identical preparation conditions. The reported values represent the average results obtained from these measurements.

Particle-size distribution was determined by sieve analysis according to PN-EN 933-1:2012 to characterize the grading of the investigated mixtures and evaluate the influence of aggregate composition on packing characteristics [52]. Standard Proctor compaction tests were performed according to PN-EN 13286-2:2010/AC:2014 to determine the moisture–density relationship, including optimum moisture content and maximum dry density of each mixture [53].

The California Bearing Ratio (CBR) test was performed according to PN-EN 13286-47:2022-04 [54] on compacted specimens prepared at the determined moisture content to evaluate the load-bearing capacity of the investigated unbound mixtures. The obtained CBR values were used for comparative assessment of the mechanical performance of the investigated compositions.

Mixture stiffness was evaluated based on deformation modulus measurements obtained from plate load testing performed under controlled laboratory conditions. Resistance to fragmentation was determined using the Los Angeles abrasion test according to PN-EN 1097-2:2020-09 [55]. Hydraulic conductivity was determined using a laboratory permeability test based on Darcy’s law to assess the drainage characteristics of the investigated mixtures. Water absorption and bulk density were determined according to standard laboratory procedures for aggregate characterization.

### 2.6. Freeze–Thaw Exposure and Field Evaluation

Durability-related behavior was evaluated through complementary natural outdoor winter exposure to assess the response of the investigated mixtures to cyclic temperature variations. The specimens were subjected to repeated temperature fluctuations involving daytime thawing conditions and nighttime freezing conditions. This field exposure was used as a complementary durability assessment under realistic climatic conditions and was not considered equivalent to a standardized laboratory freeze–thaw test. After ten freeze–thaw cycles, the samples were evaluated based on changes in compressive strength, mass loss, and visible signs of deterioration.

In a separate evaluation, resistance to repeated mechanical loading was assessed using a trial section constructed from the compacted aggregate mixture. The section was exposed to repeated truck traffic with gross vehicle masses ranging from 12 to 50 t. The observations focused on layer stability, settlement, and visible structural changes during the observation period.

### 2.7. Life Cycle Assessment

The climate-related environmental impact of the investigated mixtures was evaluated using an attributional Life Cycle Assessment (LCA) approach focused on carbon footprint quantification. The assessment was performed in accordance with the principles of ISO 14040 and ISO 14044, while the carbon footprint assessment followed the requirements of ISO 14067 [39,40,41]. The analysis adopted a cradle-to-site system boundary and considered operational processes associated with aggregate production and transportation [56].

The functional unit was defined as 1 t of finished sand–gravel–gneiss mixture, enabling direct comparison of alternative aggregate compositions and transportation scenarios.

The analyzed system included extraction and loading of raw materials, transportation of crushed gneiss from the Doboszowice quarry to the AWIDA processing facility, preparation of the final aggregate mixture, and transportation of the finished product to the construction site. The production of machinery, infrastructure construction, maintenance activities, service life, recycling, and end-of-life processes were excluded from the system boundary due to the adopted cradle-to-site approach and the focus on operational emissions related to production and transportation.

Two transportation scenarios were evaluated. The reference scenario considered production of the mixture at the AWIDA facility without additional delivery, whereas the transport scenario included delivery of the finished mixture over a distance of 40 km to the construction site.

The Life Cycle Inventory (LCI) was developed primarily from operational data collected directly at the AWIDA production facility and supplemented with emission factors obtained from scientific literature and recognized databases [57,58,59,60]. The inventory included diesel consumption during aggregate handling, fuel consumption associated with gneiss transportation, electricity consumption during mixing, and transportation of the final product.

The environmental impact was quantified using the Global Warming Potential over a 100-year time horizon (GWP100) and expressed as kg CO_2_ equivalent per functional unit. The assessment focused on greenhouse gas emissions associated with aggregate production and transportation processes [57,58,59,60].

### 2.8. Total Cost of Ownership Analysis

To complement the carbon footprint assessment, a simplified Total Cost of Ownership (TCO) analysis was performed. The analysis considered operational costs directly associated with diesel fuel consumption, electricity consumption, and transportation requirements. Fuel and electricity prices representative of Polish operating conditions were applied consistently across all analyzed scenarios.

The TCO analysis focused on production and transportation-related operational costs and was used to compare the economic implications of different aggregate compositions and transport scenarios. Capital expenditures, infrastructure costs, maintenance costs, and end-of-life costs were excluded from the assessment due to the adopted operational cost perspective.

### 2.9. Sensitivity Analysis Methodology

A sensitivity analysis was conducted to evaluate the influence of selected operational parameters on the calculated Life Cycle Assessment results and carbon footprint of the investigated mixtures. Diesel fuel consumption, electricity demand, and transportation-related parameters were varied by ±20%, while all remaining model parameters were maintained constant.

The selected variation range was applied to represent possible changes in operational conditions, equipment efficiency, and energy consumption during aggregate production and transportation. The analysis was used to identify the parameters with the greatest influence on greenhouse gas emissions and to evaluate potential strategies for reducing the carbon footprint of the investigated aggregate system.

## 3. Results

### 3.1. Freeze–Thaw Durability and Field Performance Evaluation

The durability-related behavior of the investigated mixture was evaluated using two complementary approaches: freeze–thaw exposure and repeated mechanical loading under field conditions.

#### 3.1.1. Freeze–Thaw Durability Assessment

The selected 70/30 mixture, consisting of 70% natural sand–gravel aggregate and 30% crushed gneiss, compacted at a moisture content of approximately 5%, achieved an average compressive strength of about 1500 kPa after seven days of conditioning.

**Figure 3:**
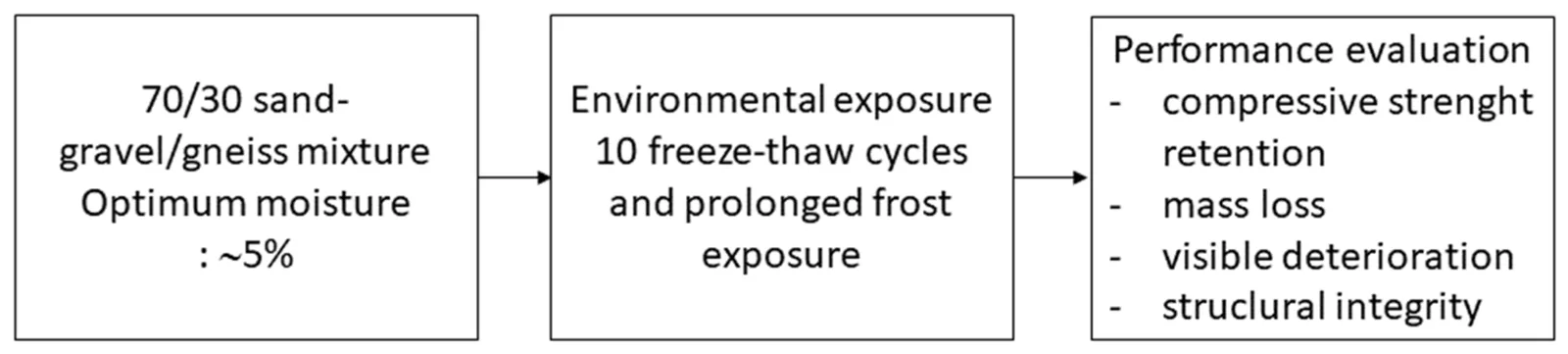
Schematic representation of the freeze–thaw exposure procedure and performance evaluation of the 70/30 sand–gravel/gneiss mixture.

After ten freeze–thaw cycles, the compressive strength remained above 1200 kPa. The average mass loss ranged from 2% to 4% during prolonged frost exposure and from 1% to 2% after freeze–thaw cycling. The freeze–thaw exposure procedure and the evaluated performance indicators are presented in Figure 3.

The compressive strength decreased to approximately 90% of the initial value after prolonged frost exposure and recovered to approximately 95% after thawing.

The 70/30 aggregate mixture maintained structural integrity under the investigated environmental conditions. During frost exposure, no visible loosening or particle disintegration was observed. Under elevated moisture conditions, the mixture remained visually homogeneous and dimensionally stable, without excessive settlement or degradation of the particle skeleton.

#### 3.1.2. Field Performance Under Repeated Mechanical Loading

The field trial was evaluated separately from the freeze–thaw exposure test. After 100 loading cycles under repeated truck traffic, no permanent deformation, rutting, or surface cracking was observed in the tested layer.

The tested layer maintained structural integrity under repeated vehicle loading during the investigated exposure period, with no visible signs of degradation.

### 3.2. Compaction Characteristics

The results of the compaction tests are summarized in Table 1. The presented values represent the average results obtained for the tested specimens. The mechanical response of the selected 70/30 sand–gravel/gneiss mixture was additionally evaluated under increasing applied loads (100, 300, and 500 kg). These loading conditions were used to assess changes in bulk density and compressive strength and were not considered as a measure of compaction energy.

The applied loads represent experimental loading conditions used to evaluate changes in bulk density and compressive strength of the 70/30 sand–gravel/gneiss mixture. They should not be interpreted as standardized compaction energy values. Compaction characteristics were evaluated separately using the Proctor test according to PN-EN 13286-2:2010/AC:2014 [53].

Increasing the applied load from 100 to 500 kg resulted in an increase in bulk density from 1.88 to 1.92 t/m^3^ and compressive strength from 1400 to 1550 kPa. The highest values of bulk density and compressive strength were obtained under the maximum applied load of 500 kg.

### 3.3. Field Observation of Utility Installation Using AWIDA Aggregate Mixture

**Figure 4:**
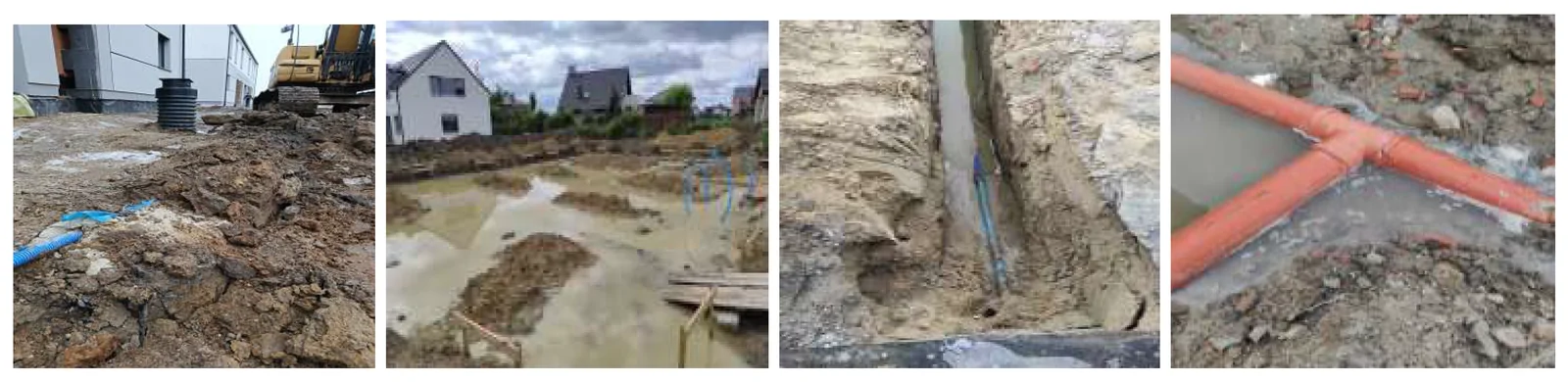
Utility trench using conventional aggregate backfill, with visible water accumulation during field inspection.

**Figure 5:**
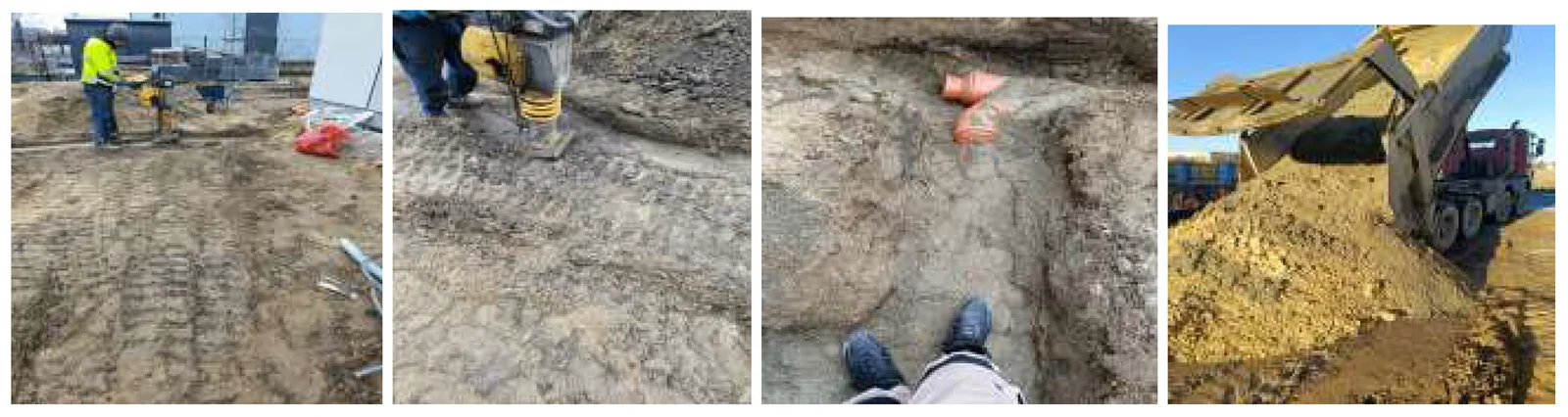
Utility installation using the compacted AWIDA 70/30 sand–gravel/gneiss mixture.

Field observations were conducted to evaluate the practical performance of the compacted AWIDA 70/30 sand–gravel/gneiss mixture under utility installation conditions. Figure 4 presents a utility trench constructed using conventional aggregate backfill, where visible water accumulation was observed during the field inspection. Figure 5 shows a utility installation constructed using the compacted AWIDA 70/30 sand–gravel/gneiss mixture.

The utility trench section containing the AWIDA 70/30 sand–gravel/gneiss mixture exhibited a homogeneous backfill structure. During the field inspection, no visible water accumulation, settlement, or deformation of the installed layer was observed.

### 3.4. California Bearing Ratio (CBR) and Deformation Modulus (E) of Sand–Gravel/Gneiss Mixtures

**Figure 6:**
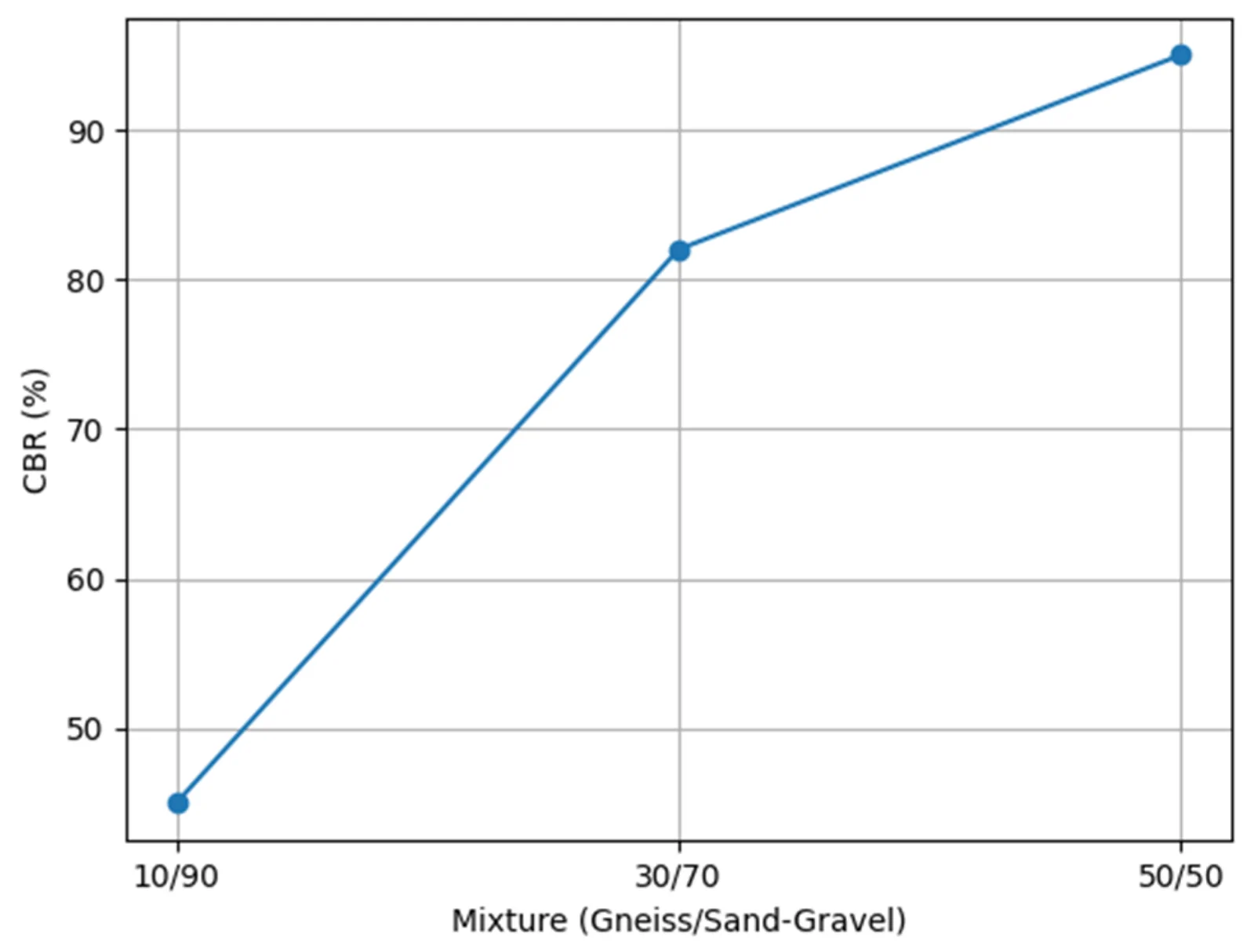
California Bearing Ratio (CBR) as a function of sand–gravel/gneiss mixture composition.

**Figure 7:**
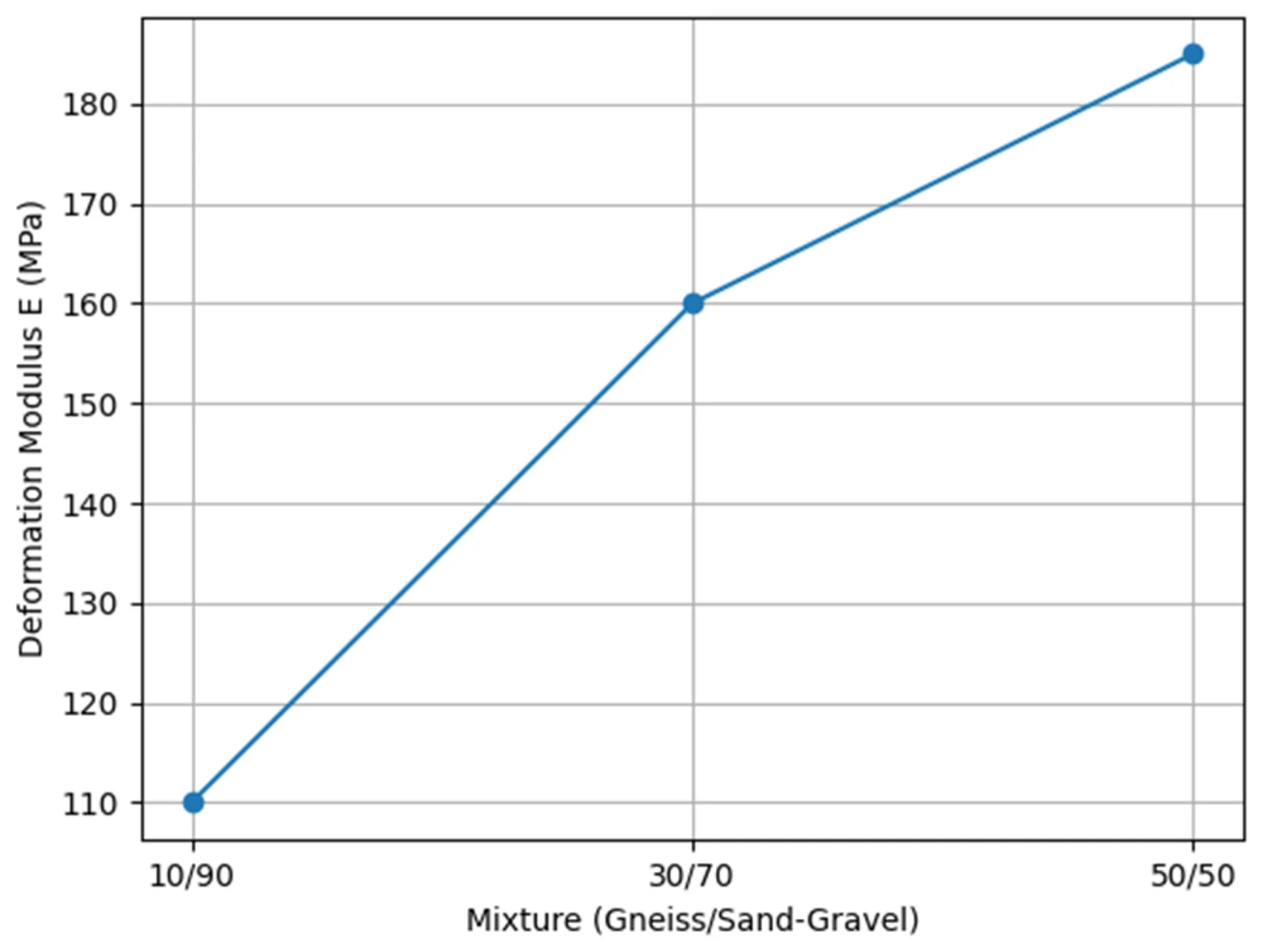
Deformation modulus (E) as a function of sand–gravel/gneiss mixture composition.

The California Bearing Ratio (CBR) and deformation modulus (E) obtained for the investigated mixtures are presented in Figure 6 and Figure 7.

The CBR and deformation modulus values for the investigated mixtures are summarized in Table 2. The CBR values increased with increasing gneiss content, from 45% for mixture M1 to 95% for mixture M3. Similarly, the deformation modulus increased from 110 MPa for M1 to 185 MPa for M3.

The results indicate that increasing the proportion of crushed gneiss in the mixture was associated with higher CBR values and deformation modulus. The highest mechanical parameters were obtained for mixture M3, containing 50% gneiss and 50% sand–gravel aggregate.

## 4. Carbon Footprint Assessment and Economic Evaluation of the Sand–Gravel/Gneiss Mixture

### 4.1. Goal and Scope of the Study

The objective of this study was to quantify the carbon footprint associated with the production of a sand–gravel/gneiss mixture produced at the AWIDA aggregate processing facility and to evaluate the influence of mixture composition and transportation distance on environmental performance and production costs. The study followed an attributional Life Cycle Assessment (LCA) approach focused on operational emissions associated with aggregate production and transportation.

The assessment was conducted according to the principles of Life Cycle Assessment described in ISO 14040 and ISO 14044 [39,40]. Since the study focused on greenhouse gas emissions, the analysis was performed in accordance with ISO 14067 for product carbon footprint quantification [41,61,62,63]. The environmental impact was expressed as Global Warming Potential over a 100-year time horizon (GWP100), reported as kilograms of carbon dioxide equivalent per functional unit (kg CO_2_ eq./FU), according to IPCC characterization factors [63,64,65,66].

The functional unit was defined as 1 t of the final sand–gravel/gneiss mixture produced at the AWIDA facility. This functional unit enables comparison of alternative mixture compositions and transportation scenarios while maintaining the same functional purpose of the product.

**Figure 8:**
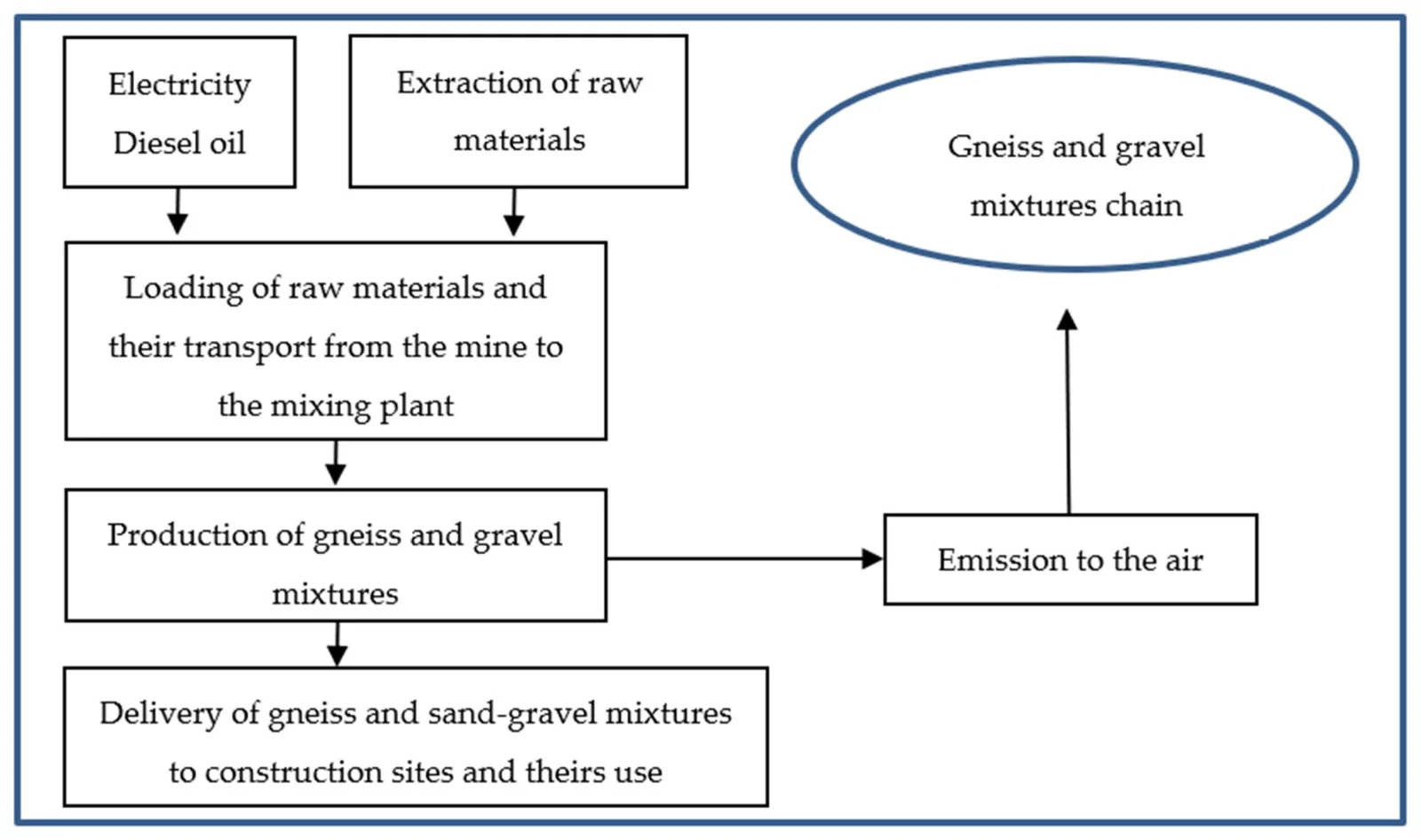
System boundaries and material flows considered in the carbon footprint assessment of the sand–gravel/gneiss mixture. The system includes raw material extraction, gneiss transportation, aggregate handling, mixing operations, and additional delivery of the final product to the construction site.

A cradle-to-site system boundary was adopted for the transport scenario, including extraction and loading of raw materials, transportation of gneiss from the Doboszowice quarry, preparation of the final mixture at the AWIDA facility, and delivery of the finished mixture to the construction site (Figure 8).

The analysis included two transportation scenarios:

- Reference scenario (Scenario I): production of the mixture at the AWIDA facility without additional delivery (0 km);
- Transport scenario (Scenario II): delivery of the final mixture to a construction site located 40 km from the processing facility.

The study excluded construction activities, service life, maintenance, recycling, and end-of-life processes because the environmental impact of bulk mineral construction materials is primarily associated with extraction, processing, and transportation stages.

No additional waste treatment processes or allocation procedures were included because the analyzed material stream was directly incorporated into the final mixture.

The environmental assessment was complemented by a Total Cost of Ownership (TCO) analysis to determine the influence of fuel consumption and transportation distance on economic performance.

### 4.2. Life Cycle Inventory (LCI)

The Life Cycle Inventory (LCI) was developed using operational data obtained from the AWIDA production process and supplemented with emission factors from scientific literature and technical sources [60,61,62].

The reference product consisted of a mixture containing 70 wt.% sand–gravel aggregate and 30 wt.% gneiss. The selected composition represents the investigated AWIDA mixture configuration and provides an appropriate balance between aggregate grading, compactability, and technical performance.

To evaluate the influence of material composition on environmental performance, three mixture variants were analyzed: 90/10 sand–gravel/gneiss, 70/30 sand–gravel/gneiss, and 50/50 sand–gravel/gneiss.

The analyzed production process included extraction, loading, transportation, and mechanical mixing. Aggregate preparation was performed using an Altrad Belle BWE-150KJ 300-L drum concrete mixer (Altrad Belle, Sheen, UK) with a rated power of 1.5 kW.

The energy demand of the mixing process was calculated based on the operating time required for the production of 1 t of mixture. Approximately 14 min were required, corresponding to two mixing cycles of approximately 7 min each.

Material handling was performed using a CAT 980K wheel loader (Caterpillar Inc., Irving, TX, USA)**.** The operational time required for loading was assumed to be 0.10 h per 1 t of material and was considered constant for all production scenarios.

The transportation of gneiss from the Doboszowice quarry to the AWIDA facility was assumed over a distance of 75 km. Transport was modelled using a Euro VI heavy-duty vehicle with an average fuel consumption of 23 L/100 km. This approach is consistent with common LCA practice for construction materials, where transport emissions are calculated based on vehicle type, payload, distance, and fuel consumption [63,64].

Fuel consumption was calculated according to equation:

FC = D × 100 FCt/100 × M/20 × 1.10

where

- FC—fuel consumption per 1 t of transported material (L/t);
- D—transport distance (km);
- FCt—average truck fuel consumption (L/100 km);
- M—transported mass (t);
- 20 t—assumed payload capacity;
- 1.10—operational correction factor.

The following emission factors were applied: diesel fuel combustion, 2.68 kg CO_2_/L; electricity consumption, 0.65 kg CO_2_ eq./kWh. The diesel emission factor represents direct combustion emissions, while electricity emissions correspond to the average emission intensity of electricity generation [63,64].

The LCI for production of 1 t of the 70/30 sand–gravel/gneiss mixture is presented in Table 3.

### 4.3. Life Cycle Impact Assessment (LCIA)

The Life Cycle Impact Assessment was limited to the climate change category because the objective of the study was to determine the carbon footprint of the aggregate mixture. This approach is consistent with ISO 14067, which provides principles for quantifying product carbon footprints based on LCA methodology [61].

The environmental impact was calculated using the IPCC GWP100 characterization approach [60,65]:

CF_total_ = CF_diesel_ + CF_electricity_ + CF_transport_

where:

- CF_diesel_—represents emissions from diesel combustion;
- CF_electricity_—represents indirect emissions associated with electricity consumption;
- CF_transport_—represents emissions associated with material transportation.

All contributions were expressed as kg CO_2_ eq. per functional unit.

For the reference 70/30 mixture, the calculated contributions were:

- Electricity consumption

0.70 kWh/t × 0.65 kg CO_2_ eq./kWh = 0.455 kg CO_2_ eq./t

- Wheel loader operation

1.00 L/t × 2.68 kg CO_2_/L = 2.680 kg CO_2_ eq./t

- Gneiss transportation

0.285 L/t × 2.68 kg CO_2_/L = 0.763 kgCO_2_ eq./t

Therefore, the total carbon footprint of the reference scenario was:

CF_total_ = 0.455 + 2.680 + 0.763

CF_total_ = 3.898 ≈ 3.90 kg CO_2_ eq./t

The calculated value represents the carbon footprint of the product at the production stage, excluding additional final product delivery.

Nitrogen oxide emissions were additionally estimated as supplementary inventory indicators. The adopted emission factors were approximately 1.64 g NOx/L diesel for wheel loader operation and 0.255 g NOx/km for Euro VI transport.

Dust emissions generated during loading and mixing were excluded from the GWP calculation because particulate matter is not included in the climate change impact category. However, dust suppression measures remain important from the perspective of local air-quality management.

### 4.4. Scenario Analysis

Additionally, a sensitivity analysis was performed to evaluate the influence of changes in diesel and electricity consumption on the final carbon footprint. A ±20% variation range was applied, representing possible operational variability of construction machinery and energy systems. To assess the influence of aggregate composition and transportation requirements on the carbon footprint of the sand–gravel/gneiss mixture, a comparative scenario analysis was performed. The analysis focused on two parameters identified in previous LCA studies as important drivers of environmental impacts for mineral construction materials: aggregate composition and transport distance [49].

The first part of the analysis evaluated the influence of gneiss content in the mixture. Although the addition of gneiss can influence particle-size distribution and mechanical properties, its use requires additional transport from an external quarry. Therefore, increasing its proportion may increase the environmental burden associated with material supply.

Three mixture compositions were investigated:

- Scenario A: 90/10 sand–gravel/gneiss;
- Scenario B: 70/30 sand–gravel/gneiss (reference scenario);
- Scenario C: 50/50 sand–gravel/gneiss.

The second part of the analysis evaluated the influence of delivery distance. Two transportation variants were considered:

- Variant I—reference production scenario: the mixture was prepared at the AWIDA facility without additional delivery (0 km);
- Variant II—construction-site delivery scenario: the final mixture was transported 40 km from the production facility.

The transport distance was selected as a representative regional supply distance for bulk construction materials. Previous studies have demonstrated that transportation can become one of the dominant contributors to the carbon footprint of aggregates due to their high mass and relatively low economic value per tonne [35,44,66].

Additionally, a sensitivity analysis was performed to evaluate the influence of variations in diesel and electricity consumption on the calculated carbon footprint. A ±20% variation range was applied to represent possible operational variability of construction machinery and energy systems.

### 4.5. Results and Contribution Analysis

#### 4.5.1. Carbon Footprint of the Reference Scenario

The carbon footprint calculated for the reference 70/30 sand–gravel/gneiss mixture without final product delivery was 3.90 kg CO_2_ eq. per tonne of product (Table 4).

This value includes emissions associated with electricity consumption, wheel loader operation, and transportation of gneiss from the Doboszowice quarry.

The contribution analysis showed that diesel fuel consumption during aggregate handling was the dominant contributor to the carbon footprint.

The results indicate that approximately 69% of the total carbon footprint was associated with wheel loader operation. This confirms observations reported in previous studies of quarrying and aggregate production systems, where fossil fuel consumption by mobile equipment represents one of the main environmental hotspots [35,36].

The high contribution of loading operations results from the considerable energy demand of heavy machinery used during extraction and material handling. Therefore, improvements in equipment efficiency, reduction in idle operating time, and transition towards lower-carbon fuels may provide substantial emission reductions.

Electricity consumption during mixing contributed approximately 12% of the total carbon footprint. This relatively low contribution is associated with the limited energy demand of aggregate mixing compared with more energy-intensive construction materials.

#### 4.5.2. Influence of Mixture Composition

The influence of mixture composition on the carbon footprint was evaluated by comparing three sand–gravel/gneiss ratios under the same production conditions (Table 5).

The lowest carbon footprint was obtained for the 90/10 mixture, with a value of 3.389 kg CO_2_ eq./t. The reduction compared with the reference scenario was mainly associated with the lower amount of transported gneiss required for mixture production. Increasing the gneiss content to 50% resulted in an increase in the carbon footprint to 4.406 kg CO_2_ eq./t. The increase of approximately 13% compared with the reference mixture demonstrates that the environmental impact of aggregate composition is strongly influenced by the origin of raw materials and associated transportation requirements.

The results demonstrate that the selection of aggregate proportions should consider not only engineering properties but also the origin and transportation requirements of individual components.

#### 4.5.3. Influence of Final Product Transportation

The introduction of a 40 km delivery distance resulted in a substantial increase in the carbon footprint of the analyzed mixture (Table 6). The additional transport generated 1.356 kg CO_2_ eq./t, increasing the total carbon footprint from 3.898 to 5.254 kg CO_2_ eq./t. The increase amounted to approximately 34.8%.

The results demonstrate that transport distance is a critical parameter influencing the environmental performance of bulk mineral products. Similar conclusions have been reported for aggregates, asphalt mixtures, and other construction materials, where transportation may become one of the dominant contributors to carbon emissions when production facilities are located far from construction sites [35,44,60].

The results indicate that reducing transport distances and maintaining regional supply chains can substantially decrease the embodied carbon of construction materials.

### 4.6. Sensitivity Analysis

A sensitivity analysis was performed to evaluate the influence of the most important operational parameters on the calculated carbon footprint. The analysis considered variations in fuel consumption, transport parameters, and energy demand. The following parameters were analyzed: diesel fuel consumption of mobile machinery, electricity consumption during mixing, and fuel consumption associated with transportation (Table 7).

A variation range of ±20% was selected to represent possible operational variability related to machine efficiency, operating conditions, and energy demand.

The sensitivity analysis confirmed that diesel consumption was the parameter with the greatest influence on the total carbon footprint. A 20% reduction in diesel consumption resulted in a decrease of approximately 0.54 kg CO_2_ eq./t compared with the reference scenario, whereas an equivalent reduction in electricity consumption resulted in only 0.09 kg CO_2_ eq./t of avoided emissions.

The results indicate that diesel-related processes represent the main opportunities for carbon footprint reduction within the analyzed system. The relatively low sensitivity to electricity consumption confirms that the mixing stage is not the main environmental hotspot of the analyzed product system.

## 5. Discussion

The results demonstrate that the performance of sand–gravel–gneiss mixtures is governed by the interaction between aggregate composition, particle morphology, and resource requirements. Increasing the proportion of crushed gneiss generally improved the engineering performance of the mixtures, which may be related to the formation of a more stable load-bearing skeleton supported by angular particles.

At the same time, higher gneiss contents increased production energy demand, transportation requirements, and consequently the carbon footprint of the mixtures. These findings highlight the importance of considering mechanical, environmental, and economic criteria simultaneously when designing aggregate mixtures for sustainable road construction.

### 5.1. Mechanical Performance and Engineering Implications

The laboratory results confirmed that crushed gneiss improved the bearing capacity of natural sand–gravel mixtures. The California Bearing Ratio (CBR) increased from 45% for the lowest-gneiss-content mixture to approximately 95% for the mixture containing 50% gneiss, while the deformation modulus increased from approximately 110 MPa to 185 MPa. These improvements may be associated with the angular morphology and rough surface texture of crushed gneiss particles, which, according to granular material theory and previous studies, can promote grain interlocking and more efficient stress transfer within the granular skeleton. However, quantitative particle-shape parameters were not determined in the present study.

The presence of approximately 10% clay fraction in the natural sand–gravel aggregate should also be considered when interpreting the obtained mechanical results. Fine particles may influence moisture sensitivity, compaction behavior, and internal pore structure of granular mixtures. However, because the clay content originated from the same AWIDA aggregate source and remained constant in all investigated mixtures, its influence was consistent across the experimental variants. Therefore, the observed differences in CBR and deformation modulus can be mainly associated with the increasing proportion of crushed gneiss and the resulting changes in granular structure.

Although the highest mechanical parameters were obtained for the 50/50 mixture, the improvement compared with the 70/30 composition should be considered together with the additional material requirements, processing demand, and environmental consequences associated with higher crushed aggregate content. Among the investigated compositions, the 70/30 mixture provided a favorable compromise between bearing capacity, stiffness, durability, and compactability. Further increases in crushed aggregate content provided additional mechanical benefits; however, these gains should be evaluated together with increased resource consumption, processing requirements, and environmental burdens. Similar observations have been reported for mechanically stabilized granular materials, where higher proportions of angular particles improve strength but may reduce workability and increase compaction effort.

Freeze–thaw durability further demonstrated that all investigated mixtures exhibited satisfactory resistance to environmental degradation under cyclic freezing and thawing conditions. Only minor mass losses were observed after freeze–thaw exposure, indicating good durability of the investigated aggregate structures. The 70/30 mixture exhibited the lowest deterioration, suggesting that the combination of rounded natural particles with angular crushed aggregate provides an internal structure capable of accommodating frost-induced stresses without visible damage. These results indicate that moderate additions of crushed gneiss may improve freeze–thaw resistance and durability characteristics without compromising constructability.

The field trial under repeated mechanical loading provided additional information regarding the short-term structural response of the investigated mixture under traffic conditions. After 100 loading cycles, no permanent deformation, rutting, or surface cracking was observed in the tested layer, indicating satisfactory resistance to the applied mechanical loading. However, these observations represent short-term field performance, and long-term monitoring under realistic traffic conditions is required for comprehensive assessment of service-life behavior.

The field observations from utility installation conditions suggest that the AWIDA 70/30 mixture may provide favorable drainage and structural stability. However, further field evaluation is required to confirm its performance under variable climatic and loading conditions.

### 5.2. Environmental Performance and Carbon Footprint

The Life Cycle Assessment demonstrated that the environmental performance of the investigated mixtures is primarily influenced by fossil fuel consumption associated with aggregate handling and transportation rather than by the mixing process itself. The reference mixture containing 70% sand–gravel and 30% crushed gneiss exhibited a cradle-to-site carbon footprint of 3.90 kg CO_2_ eq./t, which is comparable with values reported for conventional natural aggregate production systems.

Contribution analysis identified diesel consumption during wheel-loader operation as the dominant emission source, accounting for approximately 69% of the calculated carbon footprint. Transportation of crushed gneiss represented the second largest contributor (approximately 20%), whereas electricity consumption during mixing contributed only about 12%. These findings agree with previous studies showing that mobile quarry equipment and transportation are the principal environmental hotspots in aggregate production due to their intensive fuel consumption.

The influence of aggregate composition was clearly reflected in the environmental results. Increasing the proportion of crushed gneiss progressively increased greenhouse gas emissions owing to additional extraction, crushing, and transport requirements. The carbon footprint increased from approximately 3.39 kg CO_2_ eq./t for the 90/10 mixture to 4.41 kg CO_2_ eq./t for the 50/50 mixture. These differences demonstrate that improvements in mechanical performance are accompanied by measurable environmental costs, emphasizing the need to optimize aggregate composition rather than maximize crushed aggregate content.

Transportation distance proved to be another critical parameter affecting the carbon footprint of the investigated mixtures. Extending the delivery distance of the finished aggregate by 40 km increased the carbon footprint to approximately 5.25 kg CO_2_ eq./t, representing an increase of nearly 35% relative to the production stage alone. Because aggregates are characterized by high density and relatively low economic value, transportation frequently becomes one of the dominant contributors to embodied carbon. Consequently, local sourcing strategies may provide substantial environmental benefits compared with further optimization of already efficient production processes.

### 5.3. Resource Efficiency and Economic Considerations

The integration of mechanical testing with environmental and economic assessment demonstrates that aggregate composition should not be evaluated solely on the basis of strength parameters. Although the 50/50 mixture achieved the highest CBR and deformation modulus among the investigated mixtures, it also generated the highest production costs and carbon emissions due to increased crushed aggregate consumption and transportation requirements.

The Total Cost of Ownership analysis indicated that transport logistics and crushed aggregate content strongly influence overall production costs. Higher proportions of gneiss require additional quarrying, crushing, hauling, and compaction effort, increasing both operational expenditures and environmental burdens. In contrast, the 70/30 sand–gravel/gneiss mixture achieved engineering properties suitable for road construction while maintaining relatively low production costs and a moderate carbon footprint.

These results demonstrate that resource efficiency should be interpreted as achieving the required engineering performance with the minimum possible consumption of materials, energy, and transport resources.

From this perspective, the 30% gneiss mixture represented the most balanced solution within the range of investigated compositions because it combined satisfactory bearing capacity and durability with lower environmental impacts than mixtures containing higher proportions of crushed aggregate. Such a balanced approach supports sustainable resource management in infrastructure projects by reducing unnecessary consumption of high-quality rock resources.

### 5.4. Comparison with Previous Studies

The present findings are consistent with previous investigations showing that the incorporation of crushed aggregates improves the mechanical behavior of granular pavement materials by enhancing particle interlocking, stress transfer, and resistance to deformation [35,44,60]. Likewise, the carbon footprint value obtained in this study is comparable with published LCA results for natural aggregate production systems, which generally range from approximately 2 to 8 kg CO_2_ eq./t depending on extraction technology, transport distance, and energy sources.

The study also confirms observations reported in earlier quarry-based LCA investigations, where diesel-powered mobile equipment represents one of the dominant sources of greenhouse gas emissions. Compared with assessments based primarily on generic database information, the present study is based on site-specific operational data obtained directly from an operating aggregate production facility. This approach improves the reliability of emission estimates and provides results that better represent actual production conditions.

The combination of mechanical evaluation, field verification, LCA, and economic assessment provides a more comprehensive approach for selecting sustainable aggregate mixtures than assessments based solely on engineering performance.

### 5.5. Limitations and Future Research

Several limitations should be considered when interpreting the results. First, the environmental assessment was limited to the climate change category expressed as Global Warming Potential (GWP100), whereas other impact categories such as acidification, eutrophication, particulate matter formation, water consumption, and resource depletion were not evaluated. Inclusion of additional LCIA impact categories would provide a broader sustainability perspective [66].

Second, the system boundaries covered operational processes associated with aggregate extraction, processing, mixing, and transportation but excluded the manufacture and maintenance of machinery, infrastructure construction, and end-of-life scenarios. Although these processes are often reported to contribute less to total impacts than operational fuel consumption in aggregate production systems, their inclusion would enable a more comprehensive life cycle evaluation.

Finally, although laboratory testing was complemented by field observations, long-term monitoring under real traffic and climatic conditions would provide additional validation of the observed mechanical behavior and improve predictions of service life. Future research should also investigate optimization methods integrating laboratory performance, field behavior, transport logistics, regional aggregate availability, economic evaluation, and multi-criteria environmental assessment to support sustainable aggregate selection in road infrastructure projects.

## 6. Conclusions

This study evaluated the mechanical, durability, environmental, and economic performance of sand–gravel–gneiss aggregate mixtures for sustainable road construction. Laboratory testing was combined with carbon footprint assessment and Total Cost of Ownership (TCO) analysis to evaluate the influence of aggregate composition on engineering performance, environmental impacts, and production requirements. The main conclusions are as follows:

- Aggregate composition influenced the mechanical performance of the investigated mixtures. Increasing the proportion of crushed gneiss improved the California Bearing Ratio (CBR), deformation modulus, and load-bearing capacity due to the increased contribution of angular particles and improved interparticle interaction within the granular skeleton. However, higher gneiss contents were also associated with increased material requirements and greater compaction effort.
- Among the investigated compositions, the 70/30 sand–gravel/gneiss mixture provided the most balanced combination of mechanical performance, durability, and evaluated functional characteristics. This mixture achieved high bearing capacity, satisfactory freeze–thaw resistance, limited mass loss, and favorable compaction characteristics, indicating its potential suitability for unbound construction applications, subject to further validation under project-specific design and long-term field conditions.
- The environmental assessment demonstrated that greenhouse gas emissions were primarily associated with diesel consumption during aggregate handling and transportation of crushed gneiss. The reference 70/30 mixture exhibited a cradle-to-site carbon footprint of approximately 3.90 kg CO_2_ eq./t. Increasing the gneiss content or extending the transportation distance resulted in higher carbon emissions due to additional material supply and fuel consumption.
- The combined carbon footprint and economic evaluation indicated that increasing the crushed gneiss content above 30% resulted in further improvements in mechanical parameters; however, these additional mechanical benefits were accompanied by increased production costs and environmental burdens. Therefore, among the investigated mixtures, the 70/30 composition represented the most favorable compromise between engineering performance, resource demand, carbon footprint, and economic aspects within the range of compositions evaluated in this study.
- The integration of laboratory testing with carbon footprint assessment and TCO analysis provided a useful framework for evaluating aggregate mixtures by considering mechanical performance together with environmental and economic criteria.
- Overall, the results demonstrate that blending locally available natural sand–gravel aggregate with crushed gneiss within the investigated range of compositions can improve the functional performance of granular construction materials while limiting additional environmental burdens. Future research should include long-term field monitoring under actual traffic and climatic conditions, as well as expanded life cycle assessments incorporating additional environmental impact categories and regional transport optimization.

## Figures and Tables

**Table 1:** Bulk density and compressive strength of the 70/30 sand–gravel/gneiss mixture under different applied loading conditions.

Applied Load (kg)	Bulk Density (t/m^3^)	Compressive Strength (kPa)
100	1.88	1400
300	1.90	1500
500	1.92	1550

**Table 2:** California Bearing Ratio (CBR) and deformation modulus (E) of the investigated sand–gravel/gneiss mixtures (mean values obtained from two independent specimens).

Mixture	Gneiss (%)	Sand–Gravel (%)	CBR (%)	E (MPa)
M1	10	90	45	110
M2	30	70	82	160
M3	50	50	95	185

**Table 3:** Life Cycle Inventory for production of 1 t of the 70/30 sand–gravel/gneiss mixture.

Inventory Flow	Quantity	Unit	Description
Sand–gravel aggregate	0.70	t	Main aggregate fraction
Gneiss	0.30	t	Transport from Doboszowice quarry(75 km)
Electricity—concrete mixer	0.70	kWh/t	300-L mixer, rated power 1.5 kW
Diesel fuel—wheel loader	1.00	L/t	Aggregate loading and handling
Diesel fuel—gneiss transport	0.285	L/t	Transport distance: 75 km
Diesel fuel—final delivery	0/0.506	L/t	Reference scenario (0 km) and delivery scenario (40 km)

**Table 4:** Contribution analysis for the carbon footprint of the reference 70/30 sand–gravel/gneiss mixture.

Process	Carbon Footprint Contribution (kg CO_2_ eq./t)	Contribution (%)
Wheel loader operation	2.680	68.8
Gneiss transportation	0.763	19.6
Electricity consumption	0.455	11.6
Total	3.898	100

**Table 5:** Carbon footprint of different sand–gravel/gneiss compositions without final delivery.

Sand–Gravel/ Gneiss Composition	Total GWP100 (kg CO_2_ eq./t)	Gneiss Transport Contribution (kg CO_2_ eq./t)	Change Relative to Reference
90/10	3.389	0.254	−13.1%
70/30	3.898	0.763	Reference
50/50	4.406	1.271	+13.0%

**Table 6:** Contribution analysis for the 40 km final product delivery scenario.

Process	Carbon Footprint Contribution (kg CO_2_ eq./t)	Contribution (%)
Wheel loader operation	2.680	51.0
Final product delivery (40 km)	1.356	25.8
Gneiss transportation	0.763	14.5
Electricity consumption	0.455	8.7
Total	5.254	100

**Table 7:** Sensitivity analysis of the carbon footprint for the reference 70/30 sand–gravel/gneiss mixture.

Parameter	Carbon Footprint (kg CO_2_ eq./t)	Change (%)
Diesel consumption −20%	3.362	−13.8
Reference scenario	3.898	–
Diesel consumption +20%	4.434	+13.8
Electricity consumption −20%	3.807	−2.3
Electricity consumption +20%	3.989	+2.3

## Data Availability

The original contributions presented in this study are included in the article. Further inquiries can be directed to the corresponding authors.
